# PMN‐MDSCs‐induced accumulation of CD8+CD39+ T cells predicts the efficacy of chemotherapy in esophageal squamous cell carcinoma

**DOI:** 10.1002/ctm2.232

**Published:** 2020-11-10

**Authors:** Guohui Qin, Jinyan Liu, Jingyao Lian, Huanyu Zhang, Qingyang Lei, Huiyun Yang, Jingwen Shao, Xinfeng Chen, Bin Zhang, Yi Zhang

**Affiliations:** ^1^ Hospital of Zhengzhou University 1 Jianshe East Road Zhengzhou 450052 China

Dear Editor,

This study firstly demonstrated that dysfunctional CD8+CD39+ T cells are important cell subpopulations that promote esophageal squamous cell carcinoma (ESCC) progression and polymorphonuclear myeloid‐derived suppressive cells (PMN‐MDSCs) promoted CD39 expression in an IL‐6‐dependent manner. And the frequency of both PMN‐MDSCs and CD8+CD39+ T cells weresignificantly higher in chemotherapy‐resistant patient samples than thatin sensitive clinical samples. Moreover, different chemotherapy regimens and chemotherapy cycles have various effects on PMN‐MDSCs and CD8+CD39+ T cells, which provides new ideas for formulating chemotherapy combined immunotherapy programs.

Various treatment strategies have been explored for identifying and clearing tumor cells by enhancing or restoring the function of T cells, especially tumor‐infiltrating CD8+ T lymphocytes.^1^, ^2^, ^3^ However, tumor‐infiltrating CD8+ T cells have a high degree of heterogeneity. In many tumor models, bystander or exhausted T cells that are incapable of releasing cytotoxic molecules to clear tumor cells^4^ can be found at high frequencies in tumor tissues.

CD39 (ENTPD1) is an exonuclease that decomposes extracellular ATP into ADP and ADP into AMP, which is processed into adenosine by CD73 activation.^5^ Binding to the corresponding receptors, adenosine inhibits the proliferation and function of CD8+ T cells.^6^ Recently, the expression of CD39 on CD8+ T cells has been identified as a characteristic marker of tumor‐specific or exhausted CD8+ T cells.^7^, ^8^, ^9^

CD39+CD8+ T cells are a controversial group of cells because of their distribution pattern and functions. It has been demonstrated that CD39 is expressed in tumor‐specific subtype which represents the immune response to tumor antigens.^7^ However, in B16F10‐OVA tumor models, CD39+CD8+ T cells showed increased infiltration in the tumor microenvironment as tumor progressed, but exhibited exhausted characteristics with a limited secretion of IL‐2, IFN‐γ, and TNF‐α.^8^ Moreover, CD39^+^CD8^+^ T cells have also been identified as dysfunctional subsets in patients with breast cancer or melanoma. Among the reported mechanisms underlying the expression of CD39 on CD8^+^ T cells, the state of hypoxia in tumor tissues may be an important factor in promoting the continuous expression of CD39^6^. In addition, TGF‐β and IL‐27 have been shown to maintain CD39 expression in in vitro experiments.^8^ However, whether tumor microenvironment regulates the expression of CD39 and determines the phenotype or function of CD8+ T cells remains unclear.

In this study, we first investigated the distribution of CD8+ T cells and found that the frequency of CD8+ T cells in peripheral blood was significantly lower in patients with ESCC than in healthy individuals, but it was higher than tissues (Figure 1A). The clinical characteristics are shown in Table S1, and the details of antibodies are listed in Table S2. CD39, CD73, and PD‐1 were found increased in CD8+ T cells from the peripheral blood of patients with ESCC, and all three markers were highly expressed in tumor tissues (Figure 1B). Kaplan‐Meier survival analysis showed that patients with a higher infiltration of CD8+ T cells in tumor tissues had a better prognosis. Further analysis showed that patients with high frequencies of CD8+CD39+ or CD8+PD‐1+ T cells in tumor tissues had a worse prognosis, whereas the expression of CD73 on CD8^+^ T cells had no significant effect on patient survival (Figure 1C). The frequency of CD8+ T cells in the peripheral blood or tumor tissues of patients with early stage ESCC was higher than that of patients with advanced ESCC. In addition, the expression of CD39 on CD8+ T cells in advanced ESCC was significantly higher than in early stage ones (Figure 1D). Further analysis showed that CD8+CD39− T cells derived from tumor tissues secreted more IFN‐γ compared with that of CD8+CD39+ lymphocytes (Figure 1E). These results indicate that patients with advanced‐stage ESCC or worse prognosis have a lower frequency of CD8+ T cells with mostly dysfunctional subsets such as CD8+CD39+ T cells. Then the correlation analysis demonstrated that both CD39 and CD73 expression were positively correlated with PD‐1 expression in CD8+T cells (Figure 1F). These results indicate that dysfunctional CD8+CD39+ T cells are important cell subpopulations that promote ESCC progression. Therefore, elucidating the accumulation mechanisms is important for exploring new strategies of reversing the distribution of CD8^+^T cell subsets in the tumor microenvironment.

**FIGURE 1:**
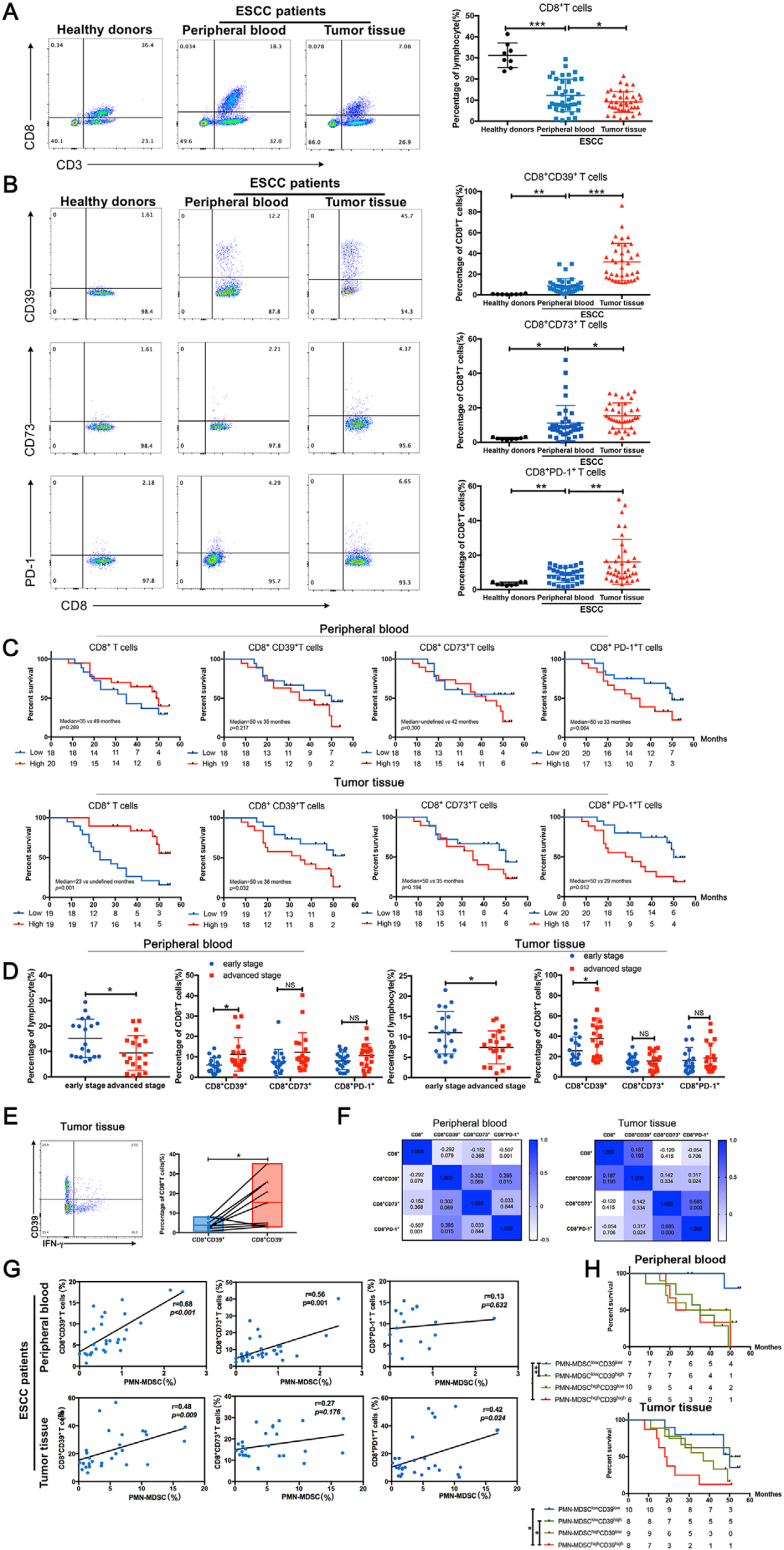
Effect of CD8+ T cells and subset distribution on the prognosis of patients with ESCC A and B. Flow cytometry was used to investigate the infiltration of CD8+ T cells and the subsets of CD8+CD39+, CD8+CD73+, and CD8+PD‐1+ T cells in healthy donors (n = 8) and patients with ESCC (n = 39). C. K‐survival analysis was used to demonstrate the correlation between the proportion of CD8+ T cells or the various subpopulations with overall survival of patients with ESCC. D. CD8+ T cell or subpopulation distribution between patients with early stage and advanced ESCC. E. Flow cytometry of the secretion capacity of IFN‐γ in CD8+CD39+ T cells and CD8+CD39‐ T cells. F, Pearson's correlation analysis was used to reveal the correlation between CD8+ T cells and various subpopulations. G, Correlation between PMN‐MDSCs (CD33+CD11b+HLA‐DR‐CD14‐) infiltration and CD8+, CD8+CD39+, CD8+CD73+, or CD8+PD‐1+ T cells in peripheral blood and tumor tissues of patients with ESCC. H, According to the infiltration of PMN‐MDSCs in peripheral blood and tumor tissues, patients with ESCC were divided into PMN‐MDSCshigh and PMN‐MDSCslow groups, and then they were further grouped according to CD8+CD39+ T cell ratio to analyze the difference in overall survival. And medium value was the cutoff points. The correlation coefficients are shown in the heat map ^*^ *P* < .05; ^**^ *P* < .01; ^***^ *P* < .001.

Our previous study showed that MDSCs significantly inhibit the proliferation and function of CD8^+^ T cells in the ESCC microenvironment. Moreover, the infiltration of PMN‐MDSCs in tumor tissues is significantly negatively correlated with the overall survival of patients with ESCC.^10^ To further explore whether MDSCs are involved in the regulation of CD39, CD73, and PD‐1 expression on CD8+ T cells, TCGA (The Cancer Genome Atlas) data of patients with ESCC showed that there was a significant positive correlation between PMN‐MDSCs (CD33) and CD39 or PD‐1 expression (Figure S1). We then conducted correlation analysis using flow cytometry data and found that the frequencies of PMN‐MDSCs were positively correlated with the accumulation of CD8+CD39+ T cells, whereas the aggregation of CD8+CD73+ T cells was significantly correlated with PMN‐MDSCs only in peripheral blood (Figure 1G). Therefore, we combined CD8+CD39+ T cells and PMN‐MDSCs to determine clinical prognosis. Patients with ESCC were first divided into a PMN‐MDSC high infiltration group and a low infiltration group, and then grouped according to the frequencies of CD8+CD39+ T cells in peripheral blood and tumor tissues. The combined analysis demonstrated that patients in the PMN‐MDSCs^high^CD39^high^ group had the worst overall prognosis (Figure 1H). Therefore, we next investigated whether PMN‐MDSCs in the ESCC microenvironment promote poor prognosis by regulating the expression pattern of CD39 on CD8+ T cells.

To investigate whether the persistence of PMN‐MDSCs can maintain CD39 expression on CD8^+^ T cells, we performed co‐incubation assays to detect CD39 expression at different time points after collecting peripheral blood samples from patients with ESCC and purifying PMN‐MDSCs or CD8^+^ T cells (Figure S2). We confirmed that CD39 expression on the surface of CD8+ T cells decreased without any intervention, whereas continuous stimulation of PMN‐MDSCs partially maintained CD39 expression (Figure 2A). There was no significant correlation between CD73 expression and PMN‐MDSC presence (Figure 2B). To investigate the mechanism by which PMN‐MDSCs increase CD39 expression on CD8+ T cells, we employed ELISA to detect the secretion of PMN‐MDSC‐related cytokines in the sera of patients with ESCC. Correlation analysis showed that IL‐6 or IL‐10 was positively correlated with PMN‐MDSC accumulation and IL‐6 was significantly negatively correlated with CD8+ T cells numbers (Figure 2C). Together with the positive correlation between CD39 and PMN‐MDSCs, we hypothesized that IL‐6 or IL‐10 is an important regulator of the immunosuppressive function of PMN‐MDSCs, which is involved in the regulation of CD39 expression. Then we added neutralizing antibodies to the co‐incubation system to eliminate the effect of IL‐6 and IL‐10, respectively, and it confirmed that inhibition of IL‐6 activity significantly reduced the PMN‐MDSC‐maintained expression of CD39 on CD8^+^ T cells, regardless of incubation time. However, there were no statistically significant differences observed between the group stimulated with PMN‐MDSCs only and IL‐10 neutralization group (Figure 2D). Furthermore, recombinant human IL‐6 significantly increased the expression of CD39, which was reversed by STAT3 inhibitor C188‐9 (Figure 2E). Immunofluorescence results also showed that PMN‐MDSCs expressed high levels of IL‐6 and that CD39 was highly expressed on CD8+ T cells (Figure 2F). After identifying IL‐6^high^ high and IL‐6^low^ groups by ELISA, overall survival analysis indicated that patients with higher levels of IL‐6 had a worse prognosis (Figure 2G).

**FIGURE 2:**
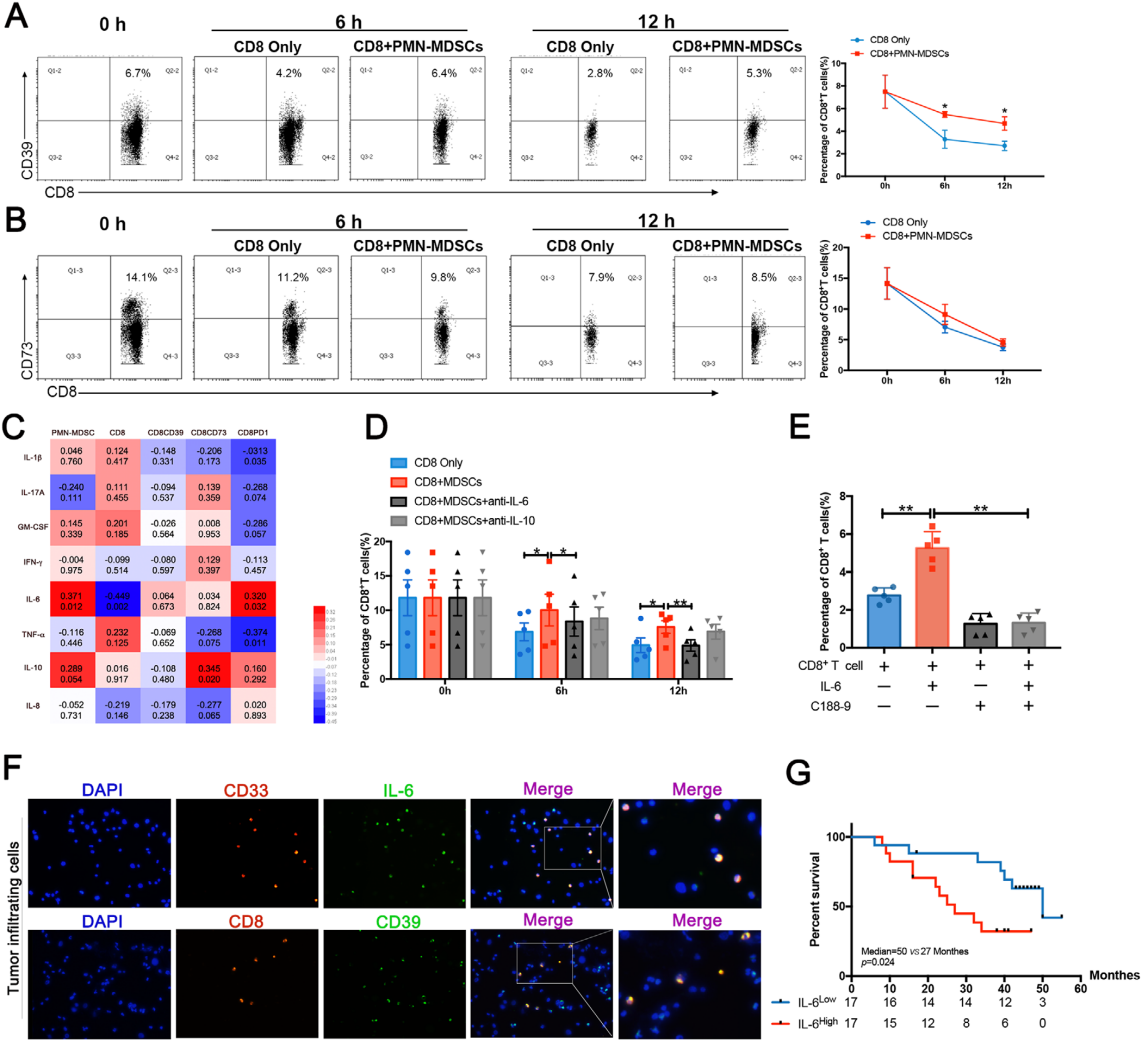
IL‐6 secreted by PMN‐MDSCs maintains CD39 expression on CD8+ T cells. A and B, CD8+ T cells and PMN‐MDSCs in the peripheral blood of patients with ESCC were sorted by magnetic beads and incubated at a ratio of 1:4. The proportion of CD8+CD39+ T cells and CD8+CD73+ T cells were detected at 0, 6, and 12 hours. C, ELISA was performed to determine the secretion of IL‐10 and other PMN‐MDSCs‐related cytokines in the sera of patients with ESCC; correlation analysis was performed with CD8+ T cell subsets. The correlation coefficients are shown in the heat map. D, Monoclonal antibodies were added to neutralize IL‐6 and IL‐10 in the co‐incubation system of PMN‐MDSCs and CD8+ T cells, after which the proportion of CD8+CD39+ T cells was detected by flow cytometry. E, IL‐6 cytokine (50 ng/mL) and STAT3 inhibitor C188‐9 (5μmol/L) were added into the culture system of CD8+ T cells isolated from ESCC patients. And CD39 expression was detected by flow‐cytometry after 12 hours. F, Single‐cell suspensions were prepared from esophageal cancer tissues for the detection of IL‐6, CD33, CD39, and CD8 by immunofluorescence. G, Patients with ESCC were divided into IL‐6high and IL‐6low groups according to IL‐6 levels in the serum, and the differences in overall survival between the two groups were analyzed^*^ *P* < .05;^**^ *P* < .01.

To assess the effect of chemotherapy on the immune status or the efficacy of immunotherapy, we obtained peripheral blood from patients 1 day prior to chemotherapy and 5 days after chemotherapy to monitor the accumulation and subset distribution of CD8+ T cells and MDSCs. Firstly, the proportion of CD8+ T cells did not change after tumor resection, but chemotherapy significantly reduced their accumulation (Figure 3A). Interestingly, tumor resection reduced the expression of CD39 on CD8+ T cells, while chemotherapy did not increase its expression (Figure 3A). Similarly, there was no significant difference in the frequencies of M‐MDSCs or PMN‐MDSCs before and after surgery in patients. However, chemotherapeutic drugs increased the accumulation of M‐MDSCs and PMN‐MDSCs in peripheral blood (Figure 3B). In addition, the secretion of PMN‐MDSCs‐related cytokines increased after chemotherapy (Figure S3). Further analysis demonstrated that patients who received one to two courses of chemotherapy had an increased frequencies of lymphocytes and decreased frequencies of CD8+CD39+ T lymphocytes, whereas those who had received three‐six courses of chemotherapy obtained a significantly reduced number of total lymphocytes or CD8+ T cells and an increased accumulation of M‐MDSC and PMN‐MDSC (Figure 3C). Taking these results into consideration, we can infer that it is better to combine immunotherapy and chemotherapy in patients receiving one‐two courses of chemotherapy. Different chemotherapy regimens also have diverse effects on the subsets’ distribution of CD8+ T cells and MDSC. The CF regimen (oxaliplatin or cisplatin combined with fluorouracil) significantly increased the number of total lymphocytes in the peripheral blood and decreased CD39 expression, but other chemotherapy drugs such as docetaxel, significantly reduced the number of total CD8+ T lymphocytes (Figure 3D). Moreover, other regimens also significantly increased PMN‐MDSC accumulation in patients with ESCC (Figure 3D). This suggests that combining CF regimens may increase the efficacy of immunotherapy in patients with ESCC. Finally, we analyzed lymphocyte distribution in the chemotherapy‐sensitive and tolerant groups and found no significant difference in the total lymphocyte count, CD8+ T cells or M‐MDSC between the two groups; however, CD8+CD39+ T cells, PMN‐MDSC, and IL‐6 in nonresponsive patients were significantly higher than in sensitive patients (Figure 3E,F). Thus, these findings can provide a reference for the choice of chemotherapy strategies for ESCC and the development of new protocols combining chemotherapy and immunotherapy.

**FIGURE 3:**
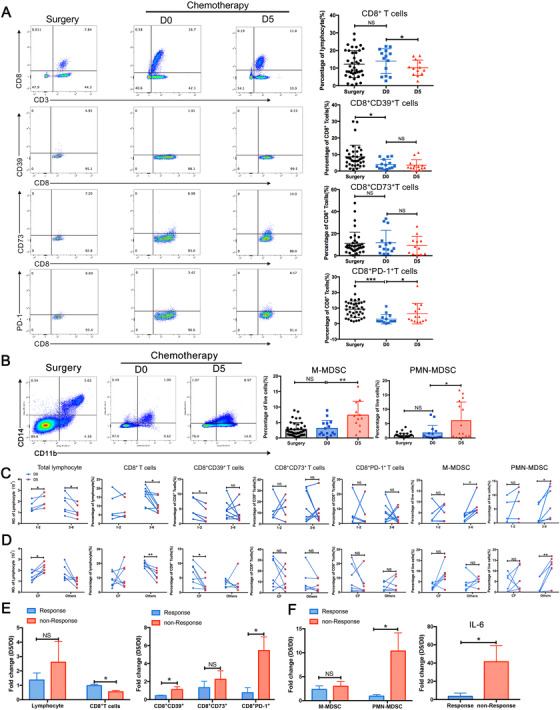
Effect of chemotherapy on the accumulation of CD8+ T cells and MDSCs. A and B, The proportions of CD8+ T cells and MDSC in patients with ESCC before and after chemotherapy (12 pairs) were analyzed by flow cytometry, and the control group was collected during surgery (n = 39). C, Patients with ESCC were divided into those who have received 1‐2 chemotherapy courses or 3‐6 courses, followed by analysis of CD8+ T cell subsets and MDSC subpopulations. D, Patients with ESCC were divided into CF or other chemotherapy treatment groups, after which changes in CD8+ T cells or MDSC were analyzed. E and F, Patients were divided into response group and nonresponse group based on tumor remission after chemotherapy. Comparative analysis of the cumulative differences in CD8+ T cells, MDSC, and IL‐6 between the two groups was performed^*^ *P* < .05;^**^ *P* < .01.

In this study, we clearly explained that PMN‐MDSC promotes the expression of CD39 in CD8^+^ T cells depending on IL‐6. The proportion of PMN‐MDSC and CD8^+^CD39^+^ T cells in chemotherapy‐resistant esophageal cancer patients was significantly higher than that in chemotherapy‐sensitive patients. On the other hand, we analyzed the effects of different chemotherapy regimens on PMN‐MDSC and CD8+CD39+ T cells, which demonstrated that CF regimen treatment significantly increased the proportion of total lymphocytes and reduced the accumulation of CD8+CD39+ T cells. However, docetaxel or gemcitabine significantly increased the ratio of PMN‐MDSC and reduced the accumulation of total CD8^+^ T cells (Figure S4).

In conclusion, this study revealed that PMN‐MDSC infiltration promotes the expression of CD39 on CD8^+^ T cells, reducing chemotherapy sensitivity and leading to poor prognosis in ESCC. Taking into consideration the effects of chemotherapy courses and regimens on MDSCs and CD8+ T cell subsets, we speculate that it will be highly beneficial to combine immunotherapy with first‐line chemotherapy regimens such as CF strategy.

## Supporting information

Supporting information

Supporting information

Supporting information

Supporting information

Supporting information

Supporting information
