# Supplementary figures and images for: PMN‐MDSCs‐induced accumulation of CD8+CD39+ T cells predicts the efficacy of chemotherapy in esophageal squamous cell carcinoma
Source: Clin Transl Med. 2020 Nov 10;10(7):e232. doi: 10.1002/ctm2.232 (PMC7654625; doi:10.1002/ctm2.232)

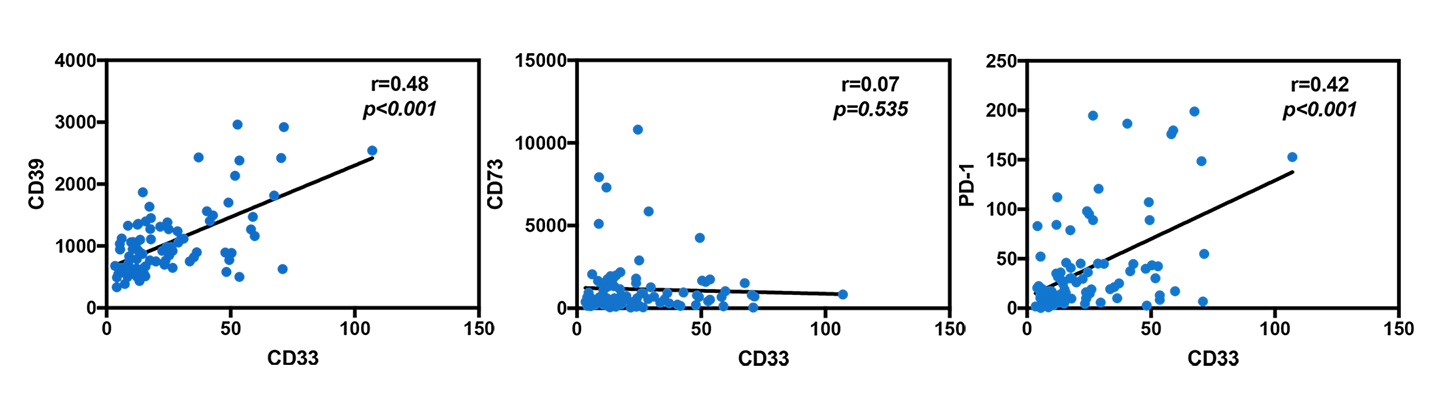

Supplement: Supplementary file 1 — Supporting information [file CTM2-10-e232-s001.tif]

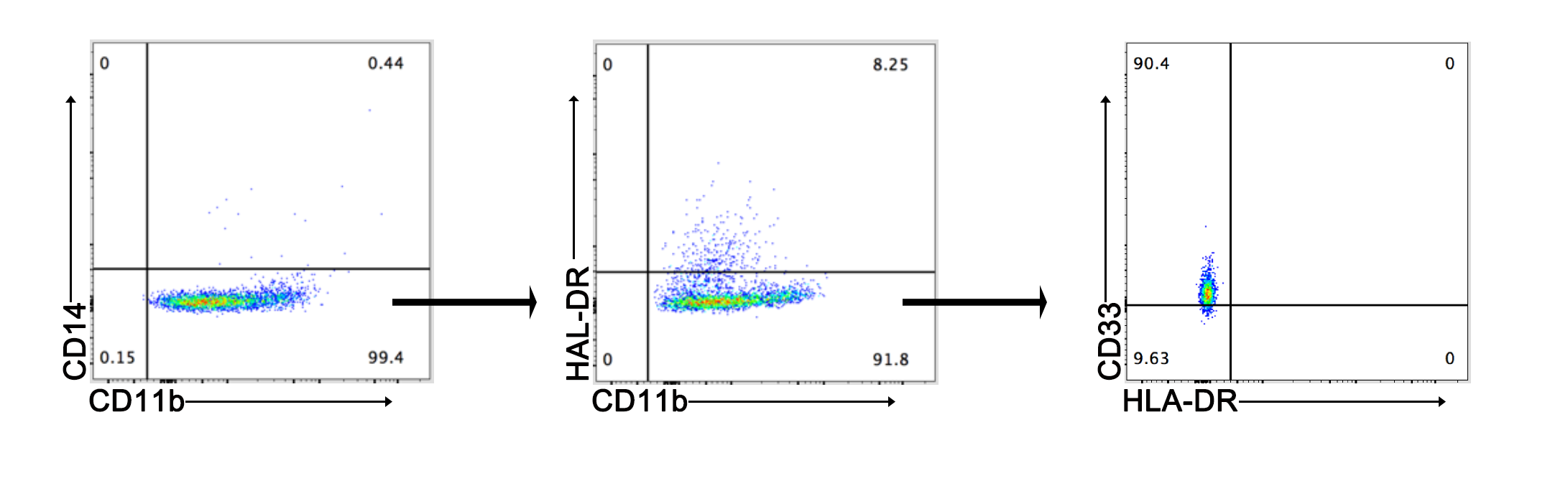

Supplement: Supplementary file 2 — Supporting information [file CTM2-10-e232-s002.tif]

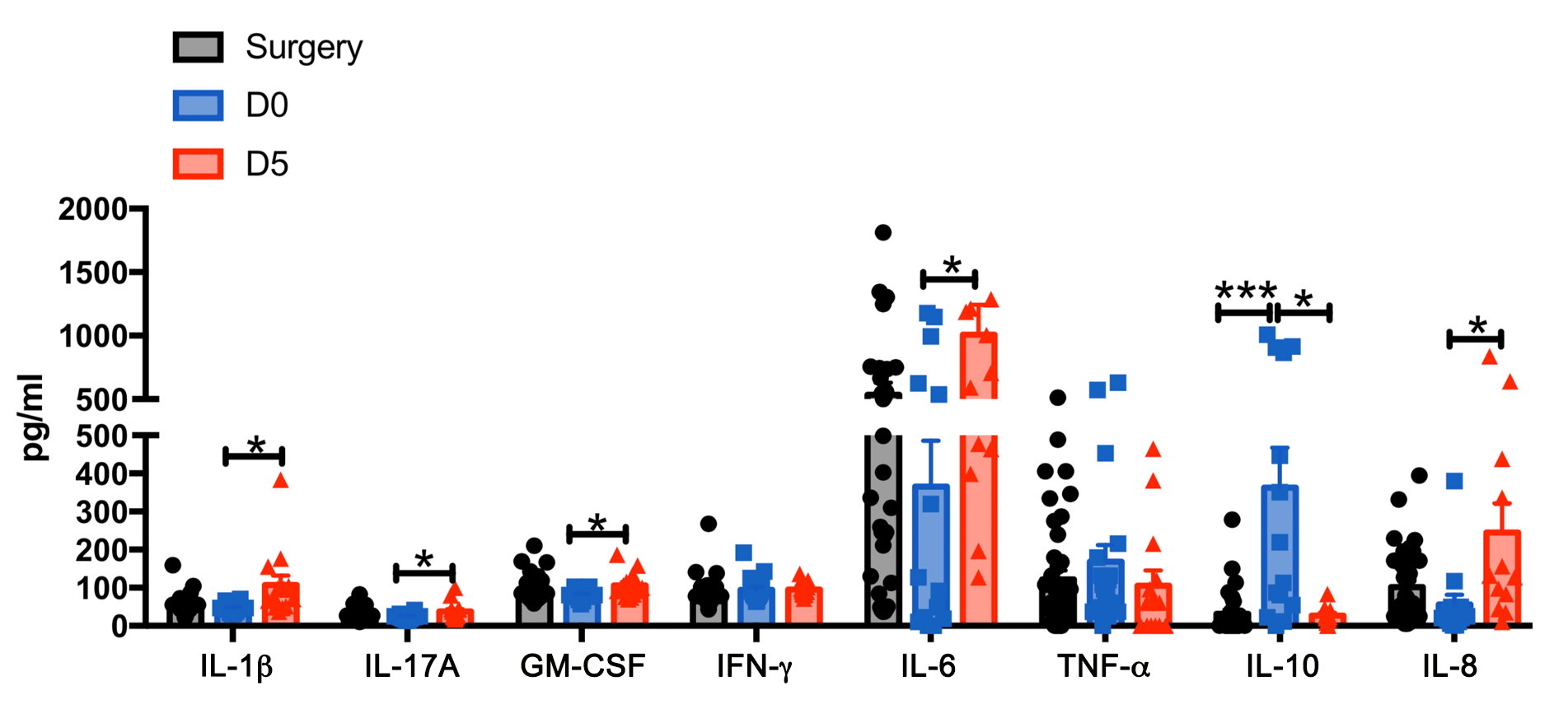

Supplement: Supplementary file 3 — Supporting information [file CTM2-10-e232-s003.tif]

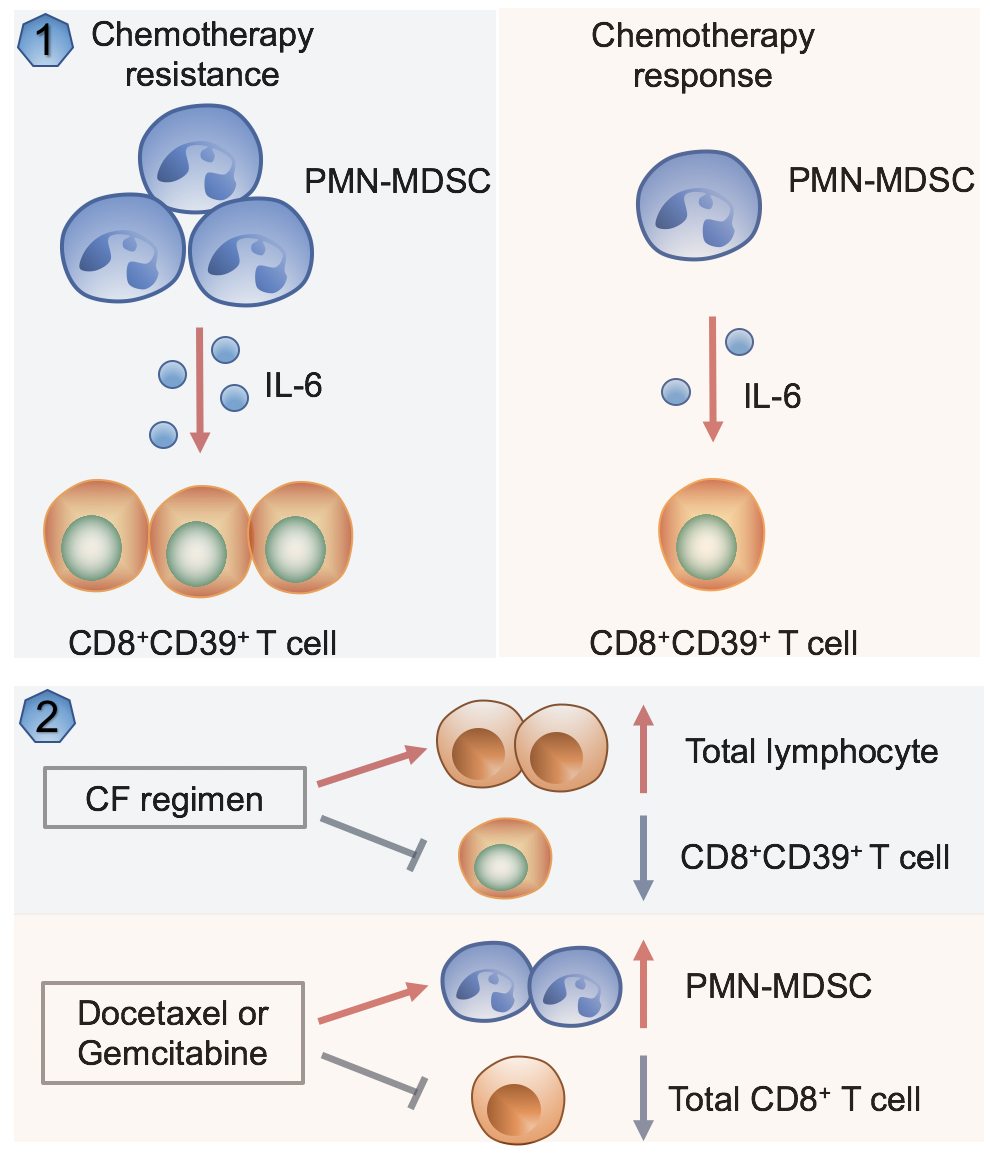

Supplement: Supplementary file 4 — Supporting information [file CTM2-10-e232-s004.tiff]
