# Supplementary material for: PMN‐MDSCs‐induced accumulation of CD8+CD39+ T cells predicts the efficacy of chemotherapy in esophageal squamous cell carcinoma
Source: Clin Transl Med. 2020 Nov 10;10(7):e232. doi: 10.1002/ctm2.232 (PMC7654625; doi:10.1002/ctm2.232)
Supplement: Supplementary file 5 — Supporting information [file CTM2-10-e232-s005.tiff]

Supplementary Table 1. Clinical pathological characteristics of patients with ESCC.

Patient Characteristics		N=39	Percentage (%)
Age at diagnosis (years old)			
	<60	17	44
	≥60	22	56
Gender			
	Male	31	79
	Female	8	21
Histological grade			
	G1	5	14
	G2	21	58
	G3	10	28
Tumor Invasion			
	T1-T2	17	44
	T3-T4	22	56
Lymph node metastasis			
	Positive	20	51
	Negative	19	49
Clinical stage number			
	Ⅰ-ⅡA	19	49
	ⅡB-Ⅳ	20	51
