# Supplementary material for: PMN‐MDSCs‐induced accumulation of CD8+CD39+ T cells predicts the efficacy of chemotherapy in esophageal squamous cell carcinoma
Source: Clin Transl Med. 2020 Nov 10;10(7):e232. doi: 10.1002/ctm2.232 (PMC7654625; doi:10.1002/ctm2.232)
Supplement: Supplementary file 6 — Supporting information [file CTM2-10-e232-s006.tiff]

Supplementary Table 2. Details of antibody and other regents in this study.

REAGENT or RESOURCE	SOURCE	IDENTIFIER
Antibodies		
PE/Cyanine7 anti-mouse/human CD11b Antibody	BioLegend	Cat #101216
APC/Cyanine7 anti-human CD14 Antibody	BioLegend	Cat #367108
PE anti-human CD33 Antibody	BioLegend	Cat #366608
FITC anti-human HLA-DR Antibody	BioLegend	Cat #307632
PE/Cyanine7 anti-human CD3 Antibody	BioLegend	Cat #317334
PerCP anti-human CD8a Antibody	BioLegend	Cat #300922
APC/Cyanine7 anti-human CD4 Antibody	BioLegend	Cat #317450
7-AAD Viability Staining Solution	BioLegend	Cat #420403
APC anti-human CD39 Antibody	BioLegend	Cat #328210
PE anti-human PD-1 Antibody	BioLegend	Cat #329906
PE anti-human IFN-γ Antibody	Proteintech	Cat # 502508
Mouse Anti-CD33 antibody	abcam	ab30371
Rabbit Anti-CD39 antibody	abcam	ab227840
Rabbit Anti-CD8 antibody	abcam	ab217344
Anti-IL-6 antibody	R&D	Cat # MAB206-SP
Anti-IL-10 antibody	R&D	Cat # MAB4176-SP
Recombinant Proteins and Critical Commercial Assays		
Recombinant Human IL-6	Peprotech	Cat # AF-200-06
Human IL-1β ELISA kit	Biolegend	Cat # 437004
Human IL-17A ELISA kit	Biolegend	Cat # 433914
Human GM-CSF ELISA kit	Biolegend	Cat # 432004
Human IL-6 ELISA kit	Biolegend	Cat # 430504
Human TNF-α ELISA kit	Biolegend	Cat # 430204
Human IL-10 ELISA kit	Biolegend	Cat # 430604
Human IL-8 ELISA kit	Biolegend	Cat # 431504
Human IFN-γ ELISA kit	Biolegend	Cat # 430101
